# Acute abdomen caused by bladder rupture attributable to neurogenic bladder dysfunction following a stroke: a case report

**DOI:** 10.1186/1752-1947-5-254

**Published:** 2011-06-29

**Authors:** Tom Mitchell, Samih Al-Hayek, Biral Patel, Fiona Court, Hugh Gilbert

**Affiliations:** 1 Department of Urology, Cheltenham General Hospital, Sandford Road, Cheltenham, Gloucestershire, GL53 7AN, UK

## Abstract

**Introduction:**

Spontaneous bladder rupture is a rare and serious event with high mortality. It is not often considered in the patient presenting with peritonitis. This often leads to delays in diagnosis. There are very few case reports of true spontaneous rupture in the literature. This is the first such reported case in which bladder rupture was attributable to neurogenic bladder dysfunction following a stroke.

**Case presentation:**

We report the case of a 67-year-old Caucasian man who presented with lower abdominal pain and a peritonitic abdomen. He had a long-term urethral catheter because of urinary retention following a previous stroke. He was treated conservatively with antibiotics before a surgical opinion was sought. Exploratory laparotomy confirmed the diagnosis of spontaneous bladder rupture. After repair of the defect, he eventually made a full recovery.

**Conclusion:**

In this unusual case report, we describe an example of a serious event in which delays in diagnosis may lead to increased morbidity and mortality. To date, no unifying theory explaining why rupture occurs has been postulated. We conducted a thorough literature search to examine the etiological factors in other published cases. These etiological factors either increase intra-vesical pressure or decrease the strength of the bladder wall. We hope that by increasing awareness of these etiological factors, spontaneous bladder rupture may be diagnosed earlier and appropriate therapy started.

## Introduction

Spontaneous bladder rupture is a rare and serious event with a mortality rate approaching 50% [1]. It is often difficult to diagnose clinically, even with the aid of increased timely access to computed tomography (CT). A number of conditions are known to predispose patients to bladder rupture, including trauma, pelvic malignancy and subsequent radiotherapy, previous bladder surgery, pregnancy, and binge alcohol drinking. Patients normally present with one of these conditions and have a short history of severe lower abdominal pain. If intra-peritoneal rupture has occurred, patients present with peritonism and blood tests consistent with acute renal failure due to the intra-peritoneal resorption of urine. Retroperitoneal rupture may be treated conservatively, but otherwise surgery is often the only modality of treatment.

### Case presentation

A 67-year-old Caucasian man presented to our hospital after an accident and emergency with a history of five hours of sudden-onset lower abdominal pain. Nine months previously he had been admitted to our hospital with a stroke due to vertebral artery dissection. He developed acute urinary retention at the time, with a residual of 550 mL of urine. He was unable to sense normal bladder filling until he experienced the pain of bladder over-distension. Previous to this he had had no lower urinary tract symptoms. His urological history included an incidental finding of an 11 mm mass upon CT in June 2009 that raised clinical suspicions of a renal cell carcinoma that was under active surveillance. His other pertinent medical history included a left inguinal hernia repair in 2008 that was initiated by using a totally extra-peritoneal approach but was converted to an open repair because of pneumoperitoneum. The patient was a recent ex-smoker, had no significant family history of urological disease, and lived independently. He was taking latanoprost and prednisolone eyedrops.

His digital rectal examination revealed a moderately enlarged prostate, and a prostate-specific antigen test returned values within normal age-related limits. He underwent anti-coagulation with warfarin as treatment for the stroke and fitted with a long-term urinary catheter that was left on free drainage.

Four months after he was fitted with the long-term catheter he had an episode of frank hematuria upon a routine catheter change. A cystoscopy was subsequently performed, which showed edematous urothelium but no focal lesions, as well as an open prostatic fossa. A trial without catheter was performed to determine whether his bladder function had recovered. This resulted in the patient's going back into urinary retention with abdominal pain. Re-catheterization drained 500 mL of urine. The catheter was replaced, and the patient was discharged with an out-patient appointment to discuss future management options.

In the interim, the patient presented to the emergency department with acute-onset lower abdominal pain. This pain was associated with diarrhea and vomiting over the preceding 24-hour period. His indwelling urinary catheter was changed without resolution of symptoms or drainage of a significant volume of urine.

An examination revealed that he was afebrile and cardiovascularly stable. His abdomen was non-distended but tense with guarding over the lower abdomen. Bowel sounds were heard. Urethral re-catheterization had drained 100 mL of urine with some light hematuria and debris in the catheter bag. Urine analysis showed 4+ blood, 4+ leukocytes, 1+ protein, and +ve nitrites. His blood tests showed neutrophilia (12.3 mL × 10^9^/mL) with a raised C-reactive protein level of 67 mg/L. He was in acute renal failure with a creatinine level of 186 mmol/L (compared with 51 mmol/L three months previously). Plain X-rays showed distended small bowel loops over the central part of the abdomen with a collapsed large bowel and no focal lung lesions or subdiaphragmatic gas. A provisional diagnosis of a urinary tract infection was made, and he was admitted under the care of the physicians. He was treated with intravenous antibiotics (piperacillin/tazobactam combination) and fluid resuscitation.

His symptoms failed to settle over the next two days, with continued loose stool, nausea, and vomiting. His urine output was good throughout (> 60 mL/hour), and his renal function normalized. However, he had regular spikes of fever reaching 38.4°C, and his inflammatory markers were raised further. A urological opinion was sought. A consultant urologist diagnosed intra-abdominal sepsis and requested general surgical involvement. CT of the abdomen and pelvis was requested.

CT showed small bowel obstruction with a transition point just above the dome of the bladder. The patient's bladder was abnormal and diffusely thickened with gas within it that tracked through the bladder dome and into the soft tissues superior and anterior to the bladder, where it was contained and formed several gas pockets that tracked toward the umbilicus. Extensive stranding was present around the dome of the bladder at the point of transition with the small bowel.

**Figure 1:**
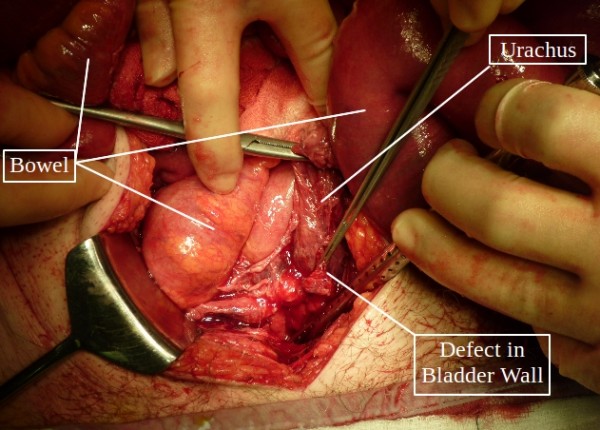
**Intra-operative view showing the defect in the bladder wall**.

The patient was taken immediately to the surgical theater for an exploratory laparotomy. A rigid cystoscopy was first performed, which showed a large defect in the dome of the bladder with a possible fistular or urachal mouth in close proximity. Biopsies of the bladder wall were taken close to the defect in the bladder dome. Laparotomy revealed a large defect in the dome of the bladder adjacent to a thickened and abnormal possible urachal remnant (Figure 1). The small bowel was dilated without any site of obstruction or bowel pathology. The bladder defect was excised with part of the wall of the bladder to allow repair. Stents, a suprapubic catheter, and two drains were placed. No obvious tumor was seen.

A histological examination of the bladder wall showed severe transmural inflammation and necrosis predominantly outside the bladder but also involving peri-vesical adipose tissue. The urothelium was reactive but unremarkable. Acute inflammation of the urachal segment extended focally to involve the mucosa, which was lost extensively. In a single section of the bladder wall, a urothelium-lined structure was identified within the lamina propria that was surrounded by smooth muscle. This may have represented a urachal remnant. No tumor, definite urachal remnant, or underlying cause of the inflammation and necrosis was identified.

Following a three-day post-operative stay in the intensive therapy unit, the patient was discharged to a general ward. His recovery was complicated by a post-operative ileus requiring total parenteral nutrition and some superficial wound dehiscence. He was then discharged to rehabilitation in a community hospital 26 days after admission and eventually fully recovered.

## Discussion

In this case report, we describe a patient who had no lower urinary tract symptoms prior to hospital admission for vertebral artery dissection. Neither an occlusive prostate nor an occlusive bladder neck was identified on cystoscopy. After his stroke, he lost all feeling of bladder filling and need to void until experiencing the pain of urinary retention. This neurological impairment continued eight months after the stroke, when a trial without catheter placement was unsuccessful. Indeed, there was no clear history of catheter blockage or discomfort before the current episode of acute abdominal pain. The long-term catheter could have predisposed him to chronic cystitis, but this is unlikely as there was no clear evidence of chronic infection on the basis of cystoscopy or histological tissue analysis.

We postulate that bladder wall dysfunction in this case was due to disturbance in neurological function because of the patient's recent stroke and possible urinary retention. Neurological dysfunction in the form of brainstem ischaemia has specifically been reported in the past as a cause of urinary retention [2]. Other acquired neurological diseases in the form of complications resulting from spina bifida [3], and diabetes mellitus [4] have been reported to cause spontaneous bladder rupture. This is the first reported case in which no clear predisposing factor could be found, but the most plausible explanation is bladder disturbance secondary to stroke. Interestingly, there are no previous reports in the literature of spontaneous bladder rupture associated with the use of long-term urinary catheters *in situ*.

A PubMed [5] search was used to help determine known etiologies for bladder rupture. We used the search terms "bladder," "rupture," and "spontaneous" in the title and abstract fields for all dates from 1980 afterward. The search returned 169 relevant cases. Most of the authors of these case reports described bladder rupture due to well-known precipitants, including trauma, congenital abnormalities, pregnancy, binge alcohol use, direct cancer spread or post-radiotherapy changes, or previous pelvic surgical interventions. Few authors have attempted to hypothesize which general etiological factors underlie bladder wall weakness [6].

Our PubMed search result was used to better classify pre-disposing factors to bladder rupture. This could help to facilitate the diagnosis of rupture in susceptible patients presenting with peritonitis. Pre-disposing pathologies may either increase intra-vesical pressure or decrease the strength of the bladder wall. Intra-vesical pressure may be increased either immediately or in the longer term. The bladder wall may be weakened locally or more generally. This categorization is shown in Table 1. Most of these pre-disposing factors may be identified on the basis of the patient's history or examination findings. We hope that this classification and the specific conditions may be used to increase awareness of risk factors for bladder rupture so that cases may be detected earlier and mortality and morbidity may be reduced.

**Table 1:** Etiological factors important in reported cases of spontaneous bladder rupture

Increase in intra-vesical pressure	Decrease in strength of bladder wall
Immediate	Localized
Blunt trauma	Trauma
Longer-term	Tumor
Alcohol-induced	Radiotherapy
Bladder outflow obstruction	Iatrogenic/post-surgery
Prostatic	Urachal cysts
Pregnancy	Diverticular
Vaginal prolapsed	Ischemia
Fecal impaction	Generalized
Neurogenic	Chronic infection/inflammation
Spina bifida	Eosinophilic cystitis
Stroke	Cytomegalovirus
Diabetes mellitus	Schistosomiasis
Psychiatric	Tuberculosis
Reduced bladder compliance	Vesical calculus
	Connective tissue disease

## Conclusion

Diagnosis of spontaneous bladder rupture can be difficult, even with increased access to CT. As in our present case, spontaneous bladder rupture can present without any of the predisposing conditions of pelvic cancer, neobladder, or trauma. Significantly, the presence of a urinary catheter does not preclude rupture. In our patient, it is likely that neuropathic bladder dysfunction secondary to a previous stroke was a major etiological factor leading to bladder rupture. This is the first such documented case of its kind.

## Consent

Written informed consent was obtained from the patient for publication of this case report and any accompanying images. A copy of the written consent is available for review by the Editor-in-Chief of this journal.

## Competing interests

The authors declare that they have no competing interests.

## Authors' contributions

TM analyzed and interpreted the patient data, reviewed the literature, and drafted the manuscript. SAH made substantial contributions to the drafting of the manuscript and revised it for intellectual content. BP and FC provided clinical information and interpretation at the time of surgery. HG made substantial contributions to the conception and design of this report and revised it critically for important intellectual content. All authors read and approved the final manuscript.
